# Research on time series prediction model for multi-factor environmental parameters in facilities based on LSTM-AT-DP model

**DOI:** 10.3389/fpls.2025.1652478

**Published:** 2025-08-18

**Authors:** Longwei Liang, Hui Shi, Zhaoyuan Wang, Shengjie Wang, Changhong Li, Ming Diao

**Affiliations:** ^1^ College of Agriculture, Shihezi University, Shihezi, China; ^2^ Research Center of Information Technology, Beijing Academy of Agriculture and Forestry Sciences, Beijing, China; ^3^ International PhD School, University of Almería, Almería, Spain; ^4^ College of Mechanical and Electrical Engineering, Shihezi University, Shihezi, China

**Keywords:** LSTM, attention mechanism, wavelet threshold denoising, multi-factor time series forecasting, environmental prediction

## Abstract

**Introduction:**

Existing facility environment prediction models often suffer from low accuracy, poor timeliness, and error accumulation in long-term predictions under multifactor nonlinear coupling conditions. These limitations significantly constrain the effectiveness of precise environmental regulation in agricultural facilities.

**Methods:**

To address these challenges, this paper proposes a novel facility environment prediction model (LSTM-AT-DP) integrating Long Short-Term Memory networks with attention mechanisms and advanced data preprocessing. The model architecture employs: (1) a Data Preprocessing (DP) module combining Wavelet Threshold Denoising (WTD) for noise elimination and Sliding Window (SW) technique for feature matrix construction; (2) an LSTM core for deep temporal modeling; and (3) an Attention Mechanism (AT) for dynamic feature weighting to enhance critical temporal feature extraction.

**Results:**

In 24-hour prediction tests, the model achieved determination coefficients (R²) of 0.9602 (temperature), 0.9529 (humidity), and 0.9839 (radiation), representing improvements of 3.89%, 5.53%, and 2.84% respectively over baseline LSTM models. Corresponding RMSE reductions were 0.6830, 1.8759, and 12.952 for these parameters.

**Discussion:**

The results demonstrate that the LSTM-AT-DP model significantly enhances prediction accuracy while effectively suppressing error accumulation in long-term forecasts. This advancement provides robust technical support for precise facility environment regulation, with particular improvements observed in humidity prediction. The integrated attention mechanism proves particularly effective in identifying and weighting critical temporal features across all measured environmental parameters.

## 1 Introduction

Facility agriculture, a vital mode of modern agricultural production, addresses the limitations imposed by external environmental conditions on crop growth by creating controlled environments through engineering and technological interventions (Gao et al., 2022). The microclimate within these facilities—encompassing temperature, relative humidity, and radiation—plays a critical role in determining crop growth, yield, and quality (Tang et al., 2022; Islam et al., 2023). Specifically, temperature fluctuations can disrupt metabolic processes, excessive humidity promotes pest and disease outbreaks, and inadequate or excessive light negatively impacts photosynthesis, leading to stunted growth or leaf damage (Jeong et al., 2020; Cheng et al., 2021). These factors exhibit multifactor nonlinear coupling, significantly complicating environmental regulation. Consequently, developing accurate predictive models for facility environments is essential to enable dynamic and precise control.

Early environmental control systems in agricultural facilities primarily depended on expert knowledge to determine optimal parameters. These systems employed manual adjustments or timed controls based on real-time environmental monitoring to maintain stable growing conditions. While this approach offers operational simplicity and has been widely adopted in facility-based production, its effectiveness is limited by significant variations in facility types and crop requirements. Such limitations often lead to delayed responses and control inaccuracies (Kow et al., 2022), causing undesirable environmental fluctuations that may disrupt plant growth and yield stability.

The advancement of sensor networks and communication technologies has led to significant improvements in agricultural environmental monitoring systems and automated control equipment. Modern facility environmental control methods have evolved from traditional manual and timed operations to setpoint-based and intelligent control approaches (Subahi and Bouazza, 2020; Maraveas and Bartzanas, 2021). While these advanced methods demonstrate superior control accuracy, enhanced plant growth performance, and reduced resource consumption compared to conventional methods, they still lack the capability for dynamic feedback control in response to environmental variations. One of the key issues that need to be addressed to realize real-time feedback control is how to achieve accurate time-by-time and day-by-day prediction of environmental factors.

The rapid development of sensor networks and communication technologies has revolutionized agricultural environmental monitoring and automated control systems. Contemporary methods of controlling facility environments have transitioned from traditional manual and timed operations to setpoint-based regulation and intelligent control strategies (Subahi and Bouazza, 2020; Maraveas and Bartzanas, 2021). Although these modern approaches exhibit significantly improved control precision, better crop growth outcomes, and enhanced resource efficiency relative to traditional methods, they remain incapable of implementing adaptive feedback control in dynamic environmental conditions. A critical research challenge for achieving real-time feedback control involves developing reliable prediction models capable of accurate hourly and daily forecasting of environmental parameters.

Current environmental prediction models can be categorized into three primary types: mechanistic models, computational fluid dynamics (CFD) models, and data-driven models (Zhang et al., 2024). Mechanistic models employ thermodynamic principles to analyze dynamic energy and mass transfer processes within agricultural facilities, characterizing system behavior through fundamental conservation laws (Zhang et al., 2020). For instance, Zhang developed an energy balance-based thermal environment model for glass greenhouses that achieved precise air temperature prediction (Zhang et al., 2020). Similarly, Liu established a transient microclimate model using thermodynamic theory to evaluate temperature and humidity variations in solar greenhouses (Liu et al., 2021). CFD models represent a specialized subset of mechanistic modeling approaches (Bournet and Rojano, 2022). A notable application by Mao demonstrated successful integration of CFD simulations with experimental measurements for comprehensive greenhouse temperature and humidity field analysis (Mao and Su, 2024).

The rapid advancement of artificial intelligence technologies has catalyzed significant progress in data-driven modeling for environmental prediction. These data-driven approaches establish predictive mapping relationships by extracting latent patterns from historical datasets, with model accuracy being critically dependent on both data quality and algorithmic architecture (Sansa et al., 2020; Mehdizadeh, 2018). A representative example is the model predictive control framework developed by Mahmood which demonstrates robust performance in facility temperature prediction under uncertain conditions, thereby validating the practical efficacy of data-driven methods in environmental forecasting applications (Mahmood et al., 2023).

In the initial development phase of data-driven approaches, researchers predominantly employed conventional machine learning models for environmental prediction, including regression models, backpropagation (BP) neural networks, recurrent neural networks (RNN), and radial basis function neural networks. These methods typically required manual preprocessing of environmental factor data (e.g., noise filtering and feature selection) prior to conducting short-term predictions through classifier training. However, the predictive performance of these early models was constrained by both the scale of input data and temporal distribution characteristics, making them inadequate for capturing the nonlinear dynamics inherent in complex environmental systems.

Addressing the intricate challenges posed by dynamically evolving environmental systems with multifactorial interactions, deep learning methods have demonstrated significant potential in environmental prediction due to their superior capability in characterizing high-order nonlinear relationships (Torres et al., 2021). Distinguished from conventional approaches, deep learning architectures not only autonomously extract high-level abstract features through multilayer nonlinear networks (Li et al., 2021), but also exhibit enhanced nonlinear approximation capacity, substantially reducing dependence on manual feature engineering. Among various deep learning models, the Long Short-Term Memory (LSTM) neural network (Hochreiter and Schmidhuber, 1997) has proven particularly effective in capturing temporal dependencies among environmental factors owing to its unique gating mechanism (Hou et al., 2023). Consequently, LSTM-based approaches have been extensively adopted for facility environment prediction. A notable application is the GCP_LSTM greenhouse climate prediction model developed by Liu, which effectively leverages LSTM networks to model nonlinear interactions among historical environmental parameters (Liu et al., 2022).

Experimental investigations have revealed that current facility environment prediction models often demonstrate inadequate accuracy and poor timeliness when handling complex nonlinear systems. Particularly as prediction time spans increase, traditional models exhibit significant accuracy degradation due to error accumulation effects. This study presents an LSTM-AT-DP based multi-step prediction framework for facility environments, focusing on temperature, humidity, and radiation parameters. Leveraging deep learning’s strengths in temporal modeling, our approach first employs a data preprocessing module to eliminate high-frequency noise from sensor-acquired time-series data, thereby mitigating error propagation risks in long-term forecasting. The denoised temporal data is then transformed into feature matrices for deep temporal pattern extraction via gated LSTM networks. Furthermore, an attention mechanism dynamically enhances feature weights at critical temporal nodes. Through multi-step prediction parameter optimization, we ultimately construct a temporally continuous prediction model. This methodology establishes a high-fidelity mapping between environmental time-series data and multi-step prediction targets, forming an intelligent deep learning-based prediction system. The proposed framework provides reliable technical support for long-cycle precision regulation strategies in controlled agricultural environments.

## 2 Materials and methods

### 2.1 Overview of the facility environment prediction model testing process

**Figure 1:**
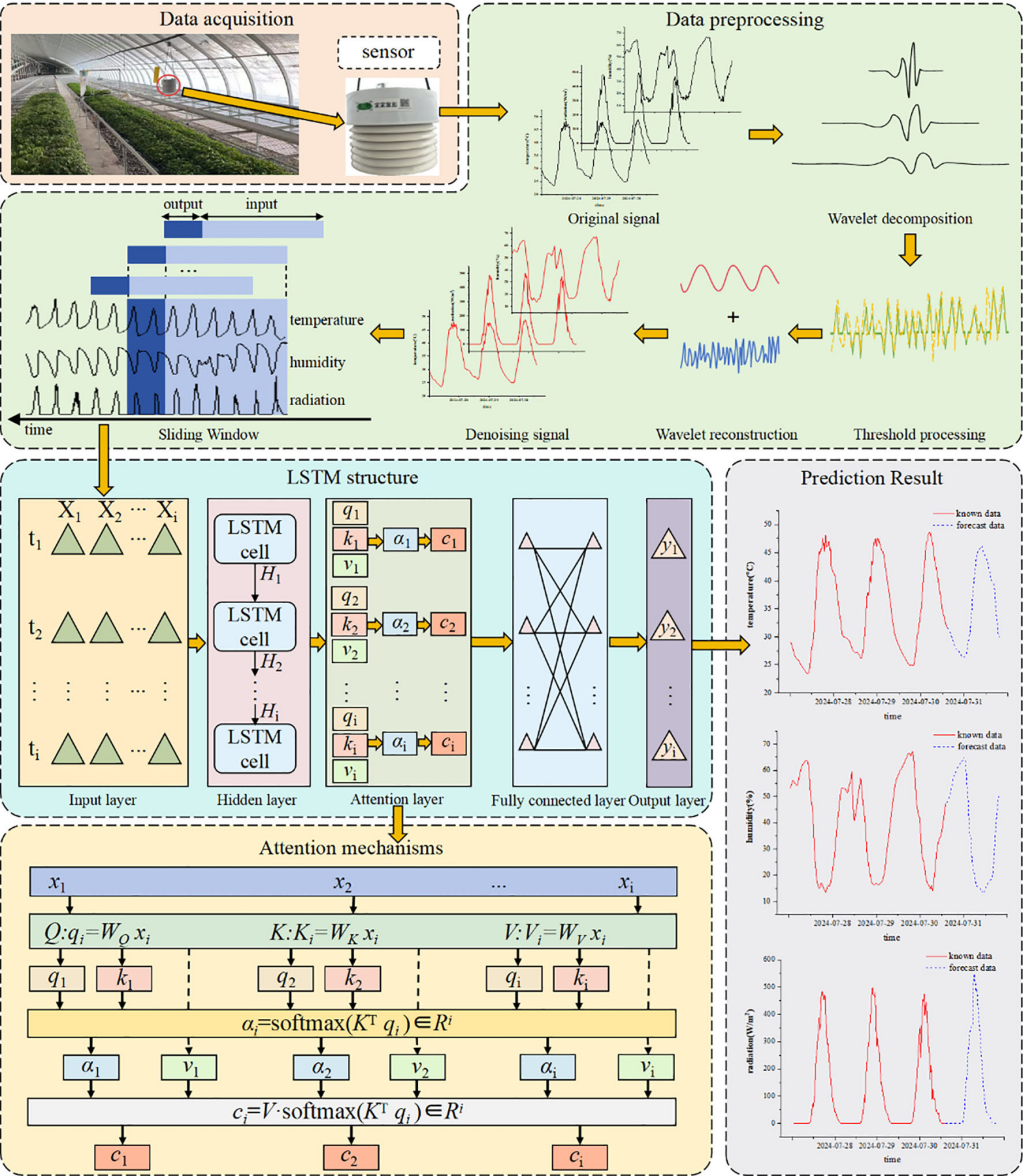
Technology roadmap.

The facility environment prediction model developed in this study addresses the complex challenge of multivariate time-series forecasting for greenhouse environmental parameters (temperature, humidity, and radiation) through advanced deep learning techniques. The framework operates through five key phases: First, distributed sensors collect real-time raw time-series data encompassing critical environmental variables including air temperature, relative humidity, and solar radiation intensity. Second, a dedicated data preprocessing stage performs noise reduction and outlier removal to enhance data quality, establishing a robust foundation for subsequent analysis. Third, the cleansed temporal data undergoes structural transformation into feature matrices for LSTM network processing, where the gated architecture provides fundamental temporal modeling capacity. Fourth, an integrated attention mechanism dynamically weights the denoised multidimensional features, enabling selective focus on critical temporal patterns while maintaining responsiveness to short-term fluctuations. Finally, the LSTM’s sophisticated gating mechanism performs deep temporal modeling and future-state prediction, complementing the attention module to achieve balanced short- and long-term dependency capture. The system outputs multi-horizon predictions (6h, 12h, and 24h) for all target environmental parameters, providing essential decision-support for precision environmental control (see **Figure 1**).

### 2.2 Overview of the experiment

The experimental study was conducted at the Xinjiang Kashgar (Shandong Shuifa) Vegetable Industry Demonstration Park (39.35°E, 76.02°N). The research facility consisted of a north-south oriented double-film double-arch greenhouse measuring 120 m in length and 18 m in width, equipped with comprehensive environmental control systems including:

1. External and internal thermal insulation screens
2. Sidewall and roof ventilation systems
3. Internal circulation fans
4. High-pressure mist cooling systems

The study utilized tomato seedlings grown in a substrate mixture of peat moss and vermiculite (2:1 v/v ratio). Experimental periods spanned from March to August 2024 and March 9 to June 26, 2025. During the early experimental phase (March), when temperatures were low, both insulation curtains were activated synergistically to maintain optimal temperatures. In the later phase (May-July), high temperatures prompted implementation of an integrated cooling strategy combining ventilation (side/top), internal air circulation, and misting systems. Throughout the study period, these precisely controlled environmental conditions were maintained to meet the developmental requirements of tomato seedlings.

### 2.3 Data acquisition

**Figure 2:**
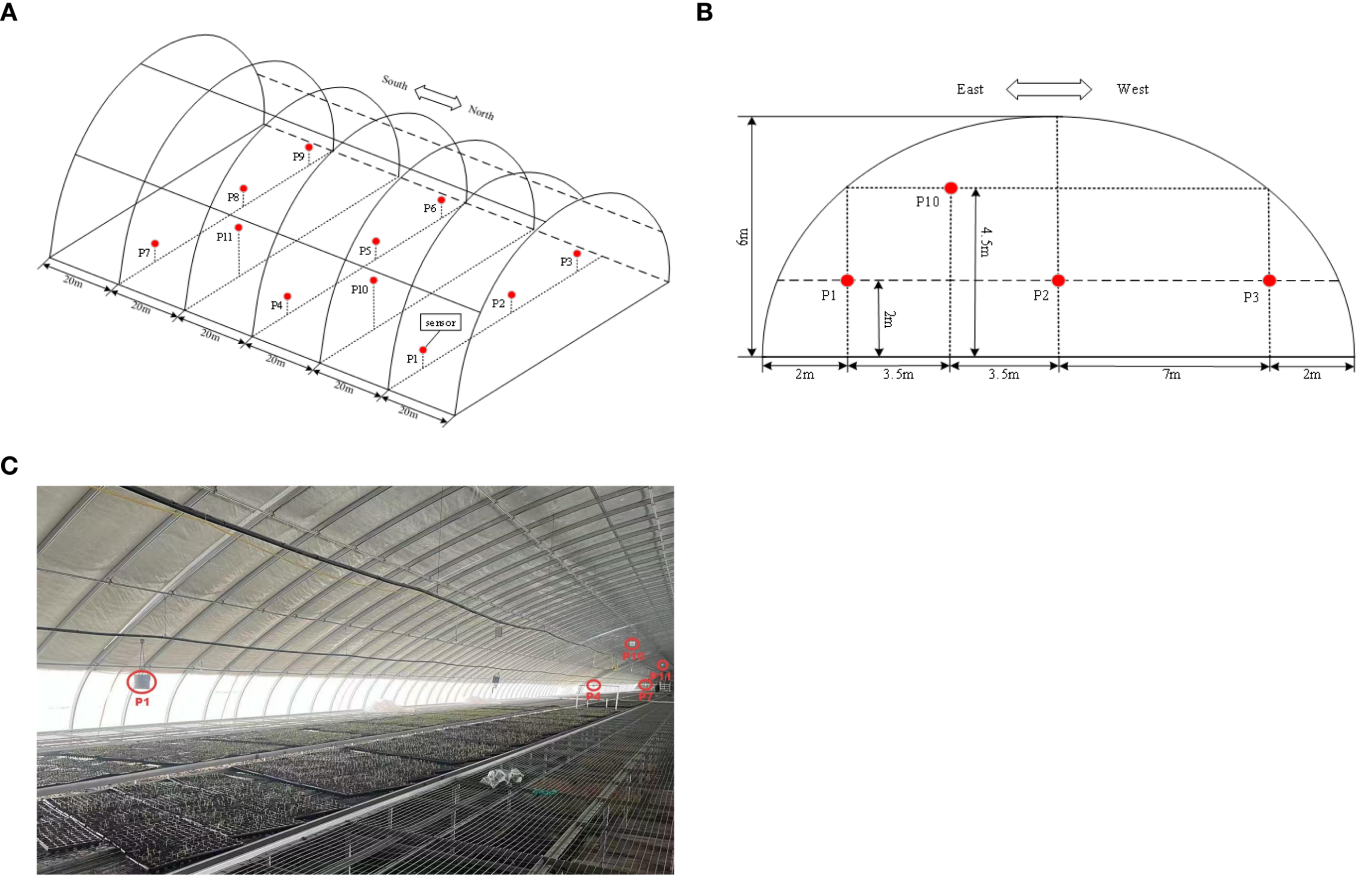
**(A)** Sensor layout diagram (front view). **(B)** Sensor layout diagram (side view). **(C)** Sensor test layout diagram.

The experimental data acquisition system employed a multi-parameter environmental monitor (Nongxin Technology, Beijing) specifically designed for greenhouse applications. The system continuously recorded three critical parameters: air temperature, relative humidity, and solar radiation intensity. Eleven monitoring nodes were strategically distributed throughout the greenhouse to ensure comprehensive spatial coverage, with their precise locations illustrated in **Figure 2**. The complete technical specifications of all deployed sensors are systematically presented in **Table 1**.

**Table 1:** Technical specifications of sensors.

Measured parameter	Model	Resolution	Accurate	Measurement range
Temperature sensor	SHT41	0.01°C	± 0.2°C	-30∼70°C
Humidity sensor	SHT41	0.01%RH	± 2%RH	0~100%RH
Radiation sensor	ISL89013	1W/m^2^	± 5%	0~1800W/m^2^

### 2.4 Data preprocessing

To address data quality issues arising from sensor acquisition and transmission anomalies, we implemented a rigorous two-step data preprocessing protocol:

Outlier detection using box-whisker plots with statistically defined thresholds, as shown in **Equations 1** and **2**:


(1) $$\begin{array}{c} U=P_{75}+1.5\times IQR \end{array}$$

(2) $$\begin{array}{c} L=P_{25}-1.5\times IQR \end{array}$$

where *U* is the upper bound, *L* is the lower bound, *P_75_* is the 75th percentile, and *P_25_* is the 25th percentile. *IQR* (interquartile range) represents the difference between the 75th and 25th percentiles (Ritter, 2023).

All identified outliers were treated as missing values.

(2) Missing Data Imputation:

For ≤5 consecutive missing points: Linear interpolation

For >5 consecutive missing points: Historical data-based imputation using nearest neighbors under identical weather conditions

This scheme is mathematically expressed by (Equation 3) (Ritter, 2023), which effectively avoids errors caused by long-span interpolation while ensuring data continuity.


(3) $$y_{t}=\{\begin{array}{c} y_{a}+\frac{\left(y_{b}-y_{a}\right)\times \left(t-t_{a}\right)}{t_{b}-t_{a}},\left(t\leq 5\right)\\ y_{h},\;\;\;\;\;\;\;\;\;\;\;\;\;\;(t>5) \end{array}\;$$

where *y_t_* is the missing value, *y_a_*, *t_a_* and *y_b_*, *t_b_* are the time and value of the first valid point before and after the missing segment, *t* is the time to be interpolated, and *y_h_* is the nearest neighbor with the same weather conditions of the historical data.

To address scale discrepancies among heterogeneous data types, all input variables were normalized to a [0,1] range using min-max normalization as formalized in (Equation 4) (Wang et al., 2024). The normalized dataset was subsequently partitioned into training, validation, and test subsets following a 7:2:1 ratio, ensuring proper model development and evaluation.


(4) $$\begin{array}{c} y=\frac{d-d_{min}}{d_{max}-d_{min}} \end{array}$$

where *d* is the original data, *d_min_* and *d_max_* are the minimum and maximum values in the original data, respectively, and *y* is the data after normalization.

## 3 Model construction

### 3.1 Long short-term memory

**Figure 3:**
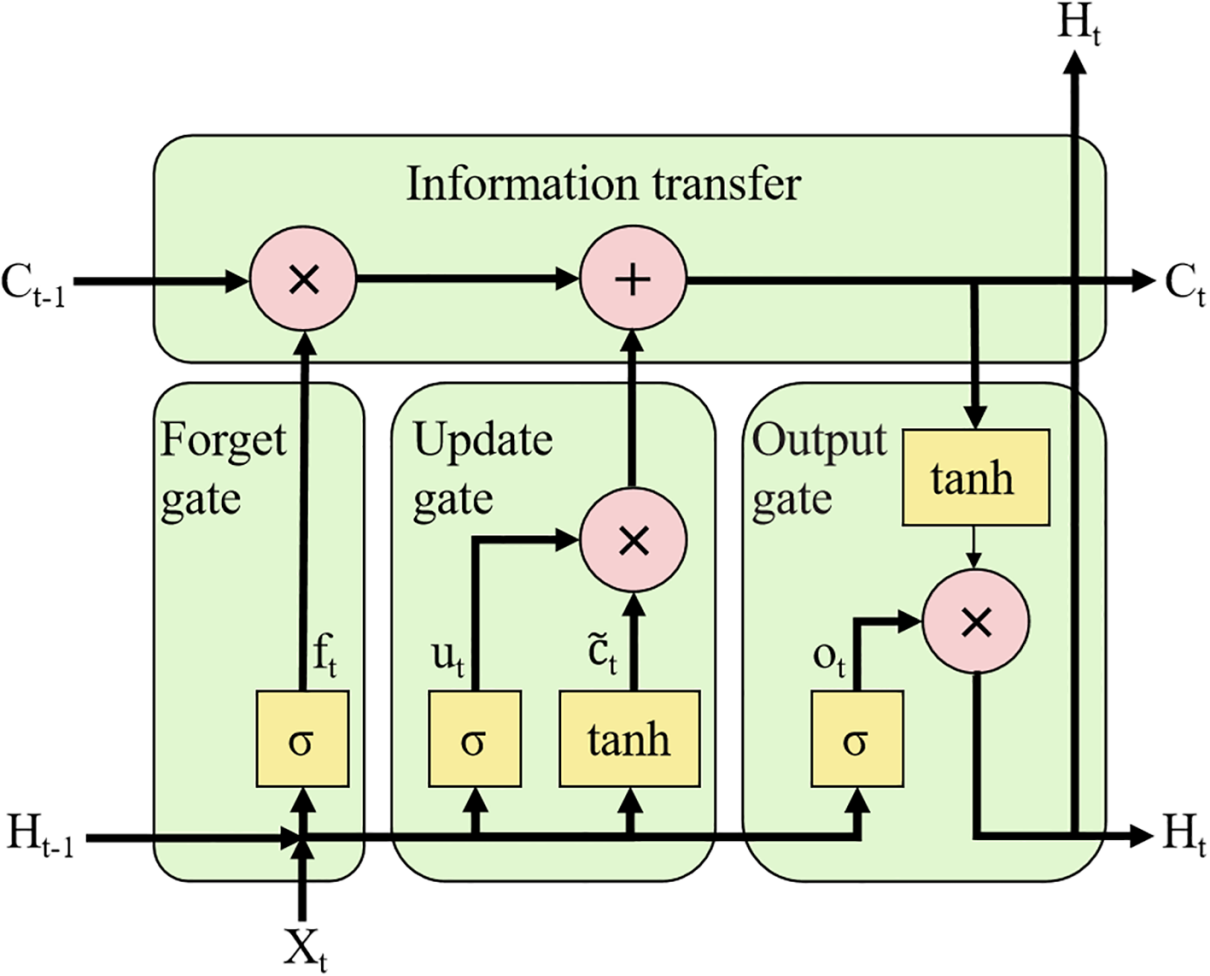
LSTM structure diagram.

Temperature, humidity, and radiation exhibit strongly nonlinear temporal characteristics (Gao et al., 2023). The LSTM architecture, as a specialized variant of recurrent neural networks (RNN), demonstrates exceptional capability in modeling such nonlinear temporal dependencies while effectively learning long-range patterns (Fan et al., 2021; Hu et al., 2025). LSTM models demonstrate superior capability in processing long-sequence data while effectively addressing the gradient vanishing issue inherent in traditional RNN architectures (Chen et al., 2020; Hong et al., 2025), as illustrated in **Figure 3**.

Therefore, the LSTM model effectively processes long-term sequential data and mitigates the gradient vanishing problem inherent in traditional RNNs. The underlying mechanism is described by the following equations (Umutoni et al., 2025):


(5) $$\begin{array}{c} f_{t}=\sigma \left(W_{f}\cdot \left[H_{t-1},X_{t}\right]+b_{f}\right) \end{array}$$

(6) $$\begin{array}{c} u_{t}=\sigma \left(W_{u}\cdot \left[H_{t-1},X_{t}\right]+b_{u}\right) \end{array}$$

(7) $$\begin{array}{c} {\tilde{c}}_{t}=tanh\left(W_{c}\cdot \left[H_{t-1},X_{t}\right]+b_{c}\right) \end{array}$$

(8) $$\begin{array}{c} C_{t}=f_{t}\cdot C_{t-1}+u_{t}\cdot {\tilde{c}}_{t} \end{array}$$

(9) $$\begin{array}{c} o_{t}=\sigma \left(W_{o}\cdot \left[H_{t-1},X_{t}\right]+b_{o}\right) \end{array}$$

(10) $$H_{t}=o_{t}tanh(C_{t})$$

where *f_t_*,*u_t_*, ${\tilde{c}}_{t}$ , $c_{t}$ , *o_t_* and *H_t_* denote the forgetting gate, updating gate, candidate cell state, current cell state, output gate, and hidden layer state, respectively; *W_f_*, *W_u_*, *W_c_*, and *W_o_* denote the weights of the forgetting gate, updating gate, cell state, and output gate; and *b_f_*, *b_u_*, *b_c_* and *b_o_* denote the bias matrices of the forgetting gate, updating gate, cell state, and output gate; tanh is the activation function and *σ* is the sigmoid activation function.

(Note: *C_t-1_* is the cell state at moment t-1; *C_t_* is the cell state at moment t; *H_t-1_* is the output at moment t-1; *H_t_* is the output at moment t.)

### 3.2 The LSTM-AT-DP model

**Figure 4:**
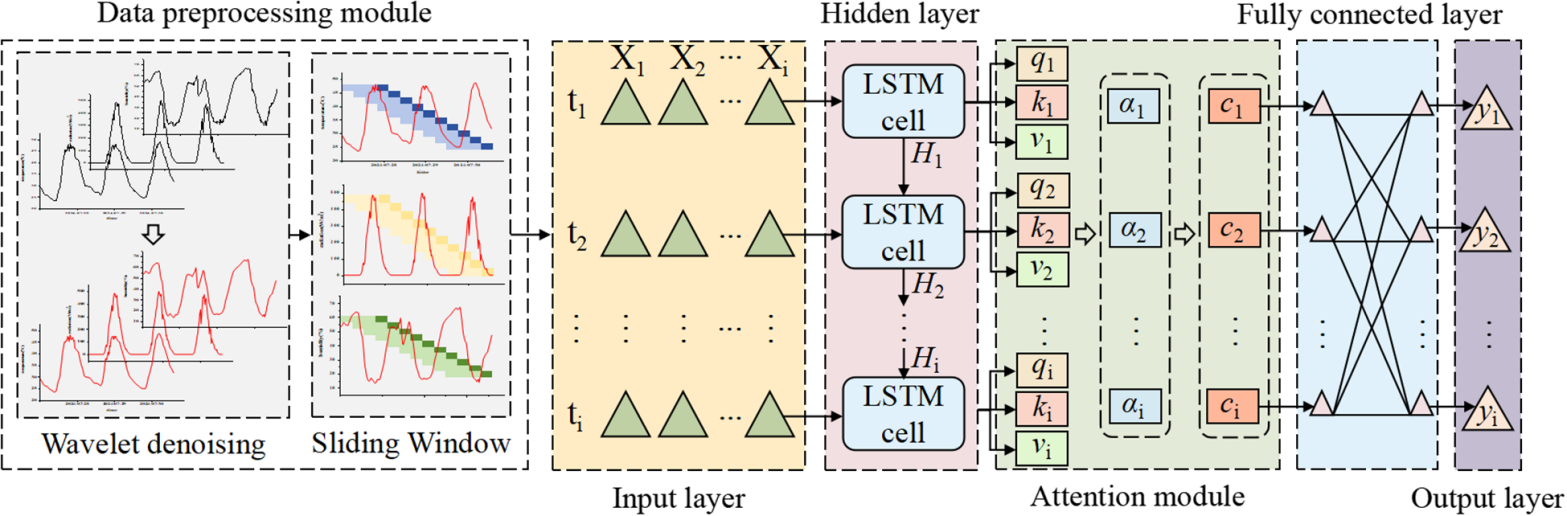
Structure diagram of the LSTM-AT-DP model.

To address the limitations of traditional models in long-term time series forecasting, particularly their declining accuracy and poor timeliness, this study proposes an LSTM-AT-DP model: Long Short-Term Memory (LSTM), Attention Mechanism Module (AT), and Data Preprocessing Module (DP). The model’s process is divided into four stages: (1) The WTD unit in the DP module first applies wavelet denoising technology to the raw time series data, significantly improving the signal-to-noise ratio; (2) the SW unit then uses sliding window technology to restructure the denoised data into a feature matrix; (3) the LSTM network extracts long-term temporal dependencies from the processed data; (4) the AT module dynamically focuses on key features and optimizes weight distribution. This model is specifically designed for facility agriculture environments and can achieve high-precision predictions of key parameters (temperature, humidity, and radiation) in greenhouse environments. **Figure 4** shows the complete architecture of this model.

The proposed LSTM-AT-DP architecture includes: data preprocessing module, input layer, hidden layer, attention mechanism module, fully connected layer, and output layer. The specific description is as follows:

1. Data preprocessing (DP) module: This module consists of two steps. First, the wavelet threshold denoising (WTD) unit performs denoising on the original multidimensional time series features. Then, the sliding window (SW) unit reorganizes the denoised sequence into a feature matrix with optimal time dependency and feeds it into the subsequent network layers.
2. Input Layer: The model takes multi-dimensional time series features as input, formatted as a 3D tensor (S, T, X) for LSTM processing, where:

S (Sample size): Sliding window length, determined by the prediction horizon:24 steps for 6-hour prediction (1 step = 15-min interval);48 steps for 12-hour prediction;96 steps for 24-hour prediction

T (Time steps): Prediction horizon (6/12/24 hours in this study).

X (Feature dimension): Three environmental parameters (temperature, humidity, and radiation).

3. Hidden Layer: Comprising multiple stacked LSTM units, this layer performs deep feature extraction and temporal dependency modeling. Through (Equations 5–10), the input information is stored in hidden states h1 to h^2^ and subsequently propagated to the next layer.
4. Attention Module: This module dynamically computes weight distributions over hidden-layer outputs to amplify the model’s focus on critical timesteps. Specifically: (Equations 14–16) derive the Query (Q), Key (K), and Value (V) vectors for each hidden state; (Equation 17) computes Q-K similarity scores, which are normalized via softmax to generate attention weights; (Equation 18) performs a weighted summation of V using these weights, producing the attention-enhanced output.
5. Fully connected layer: local feature integration and data dimension transformation of the output of the attention module.
6. Output Layer: The terminal component generates the model’s final predictions through linear transformation of the processed features, producing time-series forecasts with optimized temporal dependencies.

This study employs three evaluation metrics to assess model performance: the coefficient of determination (R²), mean absolute error (MAE), and root mean square error (RMSE). As a core indicator of regression model fitness, R² quantifies the proportion of variance in the dependent variable explainable by the model, representing the contribution of independent variables to dependent variable variation. MAE measures prediction bias, with smaller values indicating closer alignment between predicted and actual values. RMSE demonstrates particular sensitivity to peak prediction errors, where reduced values correspond to improved model accuracy. The calculation formula is as follows (**Equations 11**–**13**) (Yang et al., 2023):


(11) $$R^{2}=1-\frac{\sum_{i=1}^{N}(y^{\prime }-y)}{\sum_{i=1}^{N}(y-\bar{y})}$$

(12) $$\begin{array}{c} MAE=\frac{1}{N}\sum_{i=1}^{N}\left|y^{\prime }-y\right| \end{array}$$

(13) $$\begin{array}{c} RMSE=\sqrt{\frac{1}{N}\sum_{i=1}^{N}\left(y^{\prime }-y\right)} \end{array}$$

(Note: where *y*’ is the model predicted value, *y* is the true value, and $\bar{y}$ is the average of the true values.)

In evaluating the performance of environmental prediction models, R² and adjusted R² are key indicators for assessing model fit. As shown in **Table 2**, a systematic comparison of temperature, humidity, and radiation prediction results at three time scales (6 hours, 12 hours, and 24 hours) revealed that the Adjusted R² was slightly lower than the R² in all scenarios. This difference stems from the penalty adjustment that Adjusted R² applies to the number of features. In this study, three features were used: temperature, humidity, and radiation. When the number of features is fixed and the sample size is sufficient, the difference between the two values is less than 0.2%, indicating that the impact of model complexity on explanatory power can be ignored. Furthermore, the R² value for radiation predictions consistently exceeds 0.98, with the smallest difference from the adjusted R² value, which confirms the model’s strong explanatory power for radiation changes. Due to the stability of feature engineering, the sufficient sample size, and R²’s more intuitive interpretability in this study, it was ultimately selected as the core evaluation metric. This choice aligns with the numerical stability characteristics presented in **Table 2** and facilitates horizontal comparisons with similar studies.

**Table 2:** Comparison of R² and adjusted R².

Time	Features	R^2^	Adjusted R²	Difference
6h	Temperature	0.9723	0.9714	0.0009
	Humidity	0.9650	0.9639	0.0011
	Radiation	0.9868	0.9864	0.0004
12h	Temperature	0.9673	0.9662	0.0011
	Humidity	0.9570	0.9556	0.0014
	Radiation	0.9850	0.9845	0.0005
24h	Temperature	0.9602	0.9589	0.0013
	Humidity	0.9529	0.9513	0.0016
	Radiation	0.9839	0.9834	0.0005

### 3.3 Attention mechanism module

The AT Module dynamically weights and aggregates input information through the coordinated interaction of three vectors: Query (Q), Key (K), and Value (V) (Guo et al., 2022; Tian et al., 2022). These vectors are generated by trainable weight matrices (W_Q_, W_K_, W_V_) that project input features into respective latent spaces, rather than using random initialization. The Q vector encapsulates the current task’s informational requirements, actively retrieving relevant sequence elements. The K vector serves as a feature identifier for each input element, computing relevance scores through matching with Q. The V vector contains the actual content information that undergoes weighted aggregation based on Q and K matching results. In short, attention weights are calculated through three consecutive operations: (1) Calculate the dot product similarity between the Q vector and the K vector, (2) Standardize the operation to obtain the weights of each element of the K vector, (3) Use these normalized attention weights to perform weighted aggregation on the V vector to obtain the final result.

**Figure 5:**
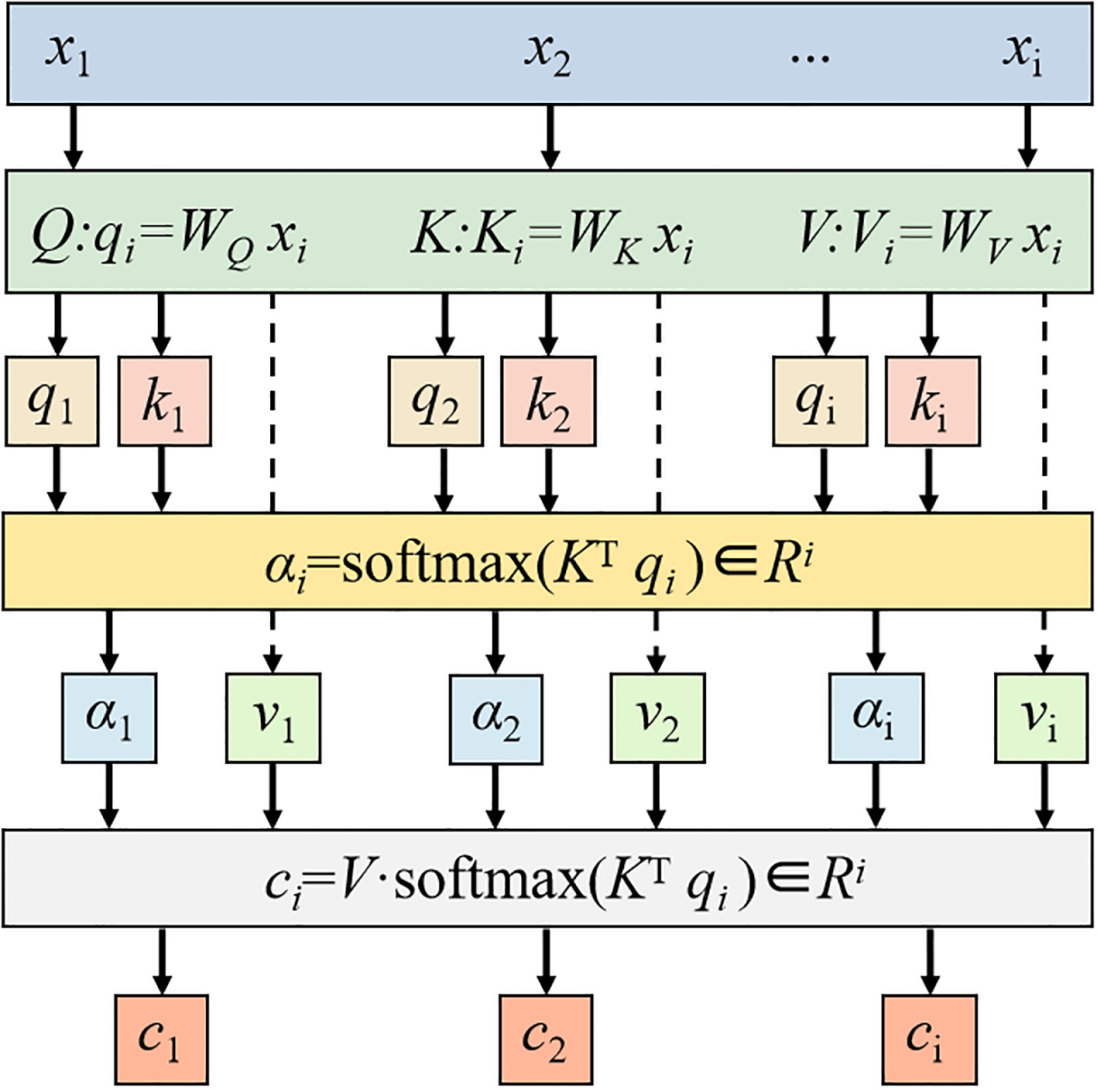
Attention module flowchart.

The specific flowchart of the attention module is shown in **Figure 5**:

The main steps in its calculation process are as follows (Zou et al., 2024):


(14) $$\begin{array}{c} Q:q_{i}=W_{Q}x_{i} \end{array}$$

(15) $$\begin{array}{c} K:K_{i}=W_{K}x_{i} \end{array}$$

(16) $$\begin{array}{c} V:KV_{i}=W_{V}x_{i} \end{array}$$

(17) $$\begin{array}{c} \alpha_{i}=softmax\left(K^{T}q_{i}\right) \end{array}$$

(18) $$\begin{array}{c} c_{i}=V\cdot softmax\left(K^{T}q_{i}\right) \end{array}$$

Where *Q* is the query vector, *K* is the key vector and *V* is the value vector; *W_Q_*, *W_K_*, *W_V_* are the parameter matrices; *q_i_* is the element of vector *Q*, *k_i_* is the element of vector *K* and *v_i_* is the element of vector *V*; *α_i_* is the attention distribution; *c_i_* is the final output; *i* is the feature order number, ranging from 1 to i.

### 3.4 Data preprocessing module

The DP module comprises two core components: (1) the Wavelet Threshold Denoising (WTD) unit that eliminates noise from raw time-series data to enhance signal-to-noise ratio, and (2) the Sliding Window (SW) unit that restructures the denoised data into equal-length continuous sequences through temporal windowing. This processing pipeline ultimately generates high-dimensional feature matrices optimized for deep feature extraction in subsequent prediction models.

#### 3.4.1 Wavelet threshold denoising unit

**Figure 6:**
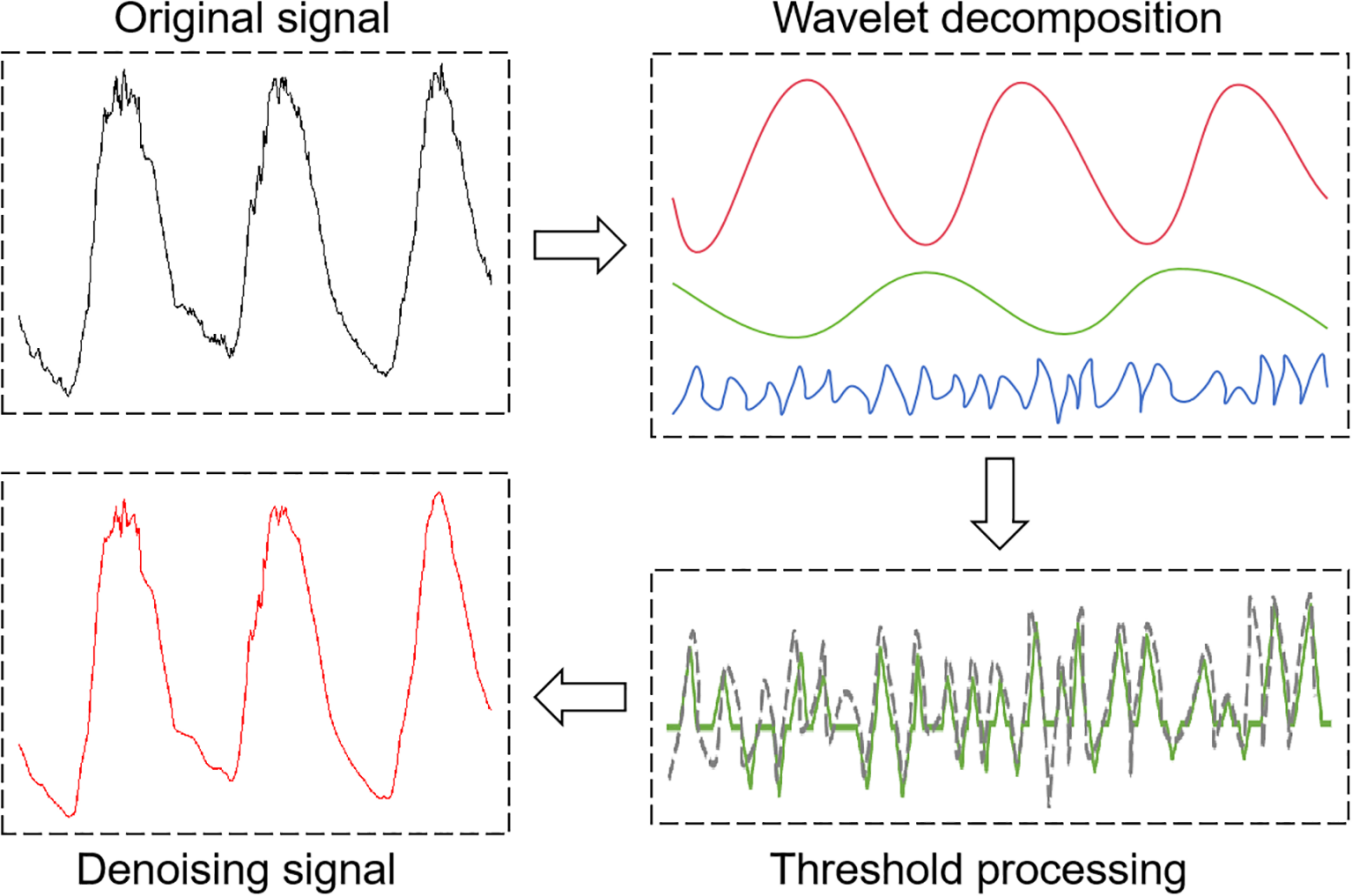
Flow diagram of the wavelet threshold denoising unit.

The WTD unit uses multiscale wavelet analysis to suppress noise through coefficient threshold processing. The process involves: decomposing the input signal into multi-band wavelet coefficients, applying optimal threshold processing to high-frequency (noise-dominated) coefficients, and reconstructing the signal from the processed coefficients (Dong et al., 2024; Li et al., 2025). WTD unit demonstrates excellent denoising capabilities due to its computational efficiency and adaptive properties (Jiang et al., 2025). As shown in **Figure 6**, noise reduction performance critically depends on three fundamental parameters: (i) choice of wavelet basis functions, (ii) threshold optimization strategy, and (iii) number of decomposition levels (Wu et al., 2024). These parameters collectively form the core foundation of the entire data preprocessing pipeline.

This study selected the sym4 wavelet for analysis and denoising to address the significant periodicity (e.g., diurnal cycles and seasonal variations) exhibited by facility environmental parameters (e.g., temperature, humidity, and radiation), multi-parameter coupling effects, and non-stationarity caused by noise artifacts resulting from control operations (e.g., ventilation and shading). The sym4 wavelet possesses approximate symmetry and excellent time-frequency locality. Experimental results show that, when set to a decomposition level of 2 and using the minimax threshold strategy, the Sym4 wavelet outperforms other wavelet basis functions in terms of noise reduction performance (ASNR = 40.23 dB, RMSE = 0.0681, and CC = 0.9999). This selection improves the signal-to-noise ratio and preserves more of the original signal’s features, providing a reliable foundation for subsequent signal processing. The following mathematical formulas (**Equations 19**, **20**) (Dong et al., 2024) illustrate this point:


(19) $$\begin{array}{c} cA_{L}\left[n\right]=\sum_{k}x\left[k\right]\cdot \phi_{L,n}\left(k\right) \end{array}$$

(20) $$\begin{array}{c} cD_{L}\left[n\right]=\sum_{k}x\left[k\right]\cdot \psi_{L,n}\left(k\right) \end{array}$$

where *cA_L_* [*n*] is the jth layer approximation coefficient (low frequency part) and *cD_L_* [*n*] is the jth layer detail coefficient (high frequency part); *x* is the original signal, *ψ* is the wavelet function, and *ϕ* is the scale function.

The soft thresholding method was selected for processing facility environmental data due to its superior performance characteristics: continuous shrinkage properties that maintain signal smoothness, effective suppression of low-amplitude noise components, robust preservation of transient features, and optimal output smoothness. These advantages make it particularly suitable for analyzing non-stationary environmental parameters. The mathematical expression for this thresholding method is shown in **Equation 21** (Li et al., 2025):


(21) $$\eta_{s}\left(\text{a},\text{λ}\right)=\{\begin{array}{c} a-\lambda ,\;a>\lambda \\ 0,\;\left|a\right|\leq \lambda \\ a+\lambda ,\;a<-\lambda \end{array}$$

where *a* is the wavelet coefficient and is the wavelet threshold *λ*.

The process of restoring the processed wavelet coefficients to the original signal is given by **Equation 22** (Jiang et al., 2023):


(22) $$\begin{array}{c} x_{r}=\sum_{k}cA_{L}\left[k\right]\cdot \phi_{L,k}\left(t\right)+\sum_{j=1}^{L}\sum_{k}cD_{j}\left[k\right]\cdot \psi_{j,k}\left(t\right) \end{array}$$

where *x_r_* is the reconstructed signal.

The reconstructed denoised signal *x_r_* is used as an input to the sliding window, whereby the sliding window unit generates the input data required for the prediction model.

#### 3.4.2 Sliding window unit

The monitoring and prediction of facility environmental parameters (temperature, humidity, solar radiation) constitute a characteristic multivariate time series forecasting problem. This problem presents two fundamental challenges: (1) strong temporal dependencies (e.g., thermal inertia effects, radiative accumulation), and (2) complex cross-variable dynamics (e.g., humidity-temperature coupling, radiation-driven cross-effects). Naive approaches using instantaneous independent observations fail to capture the temporal continuity of environmental evolution, leading to inaccurate predictions of future facility states.

**Figure 7:**
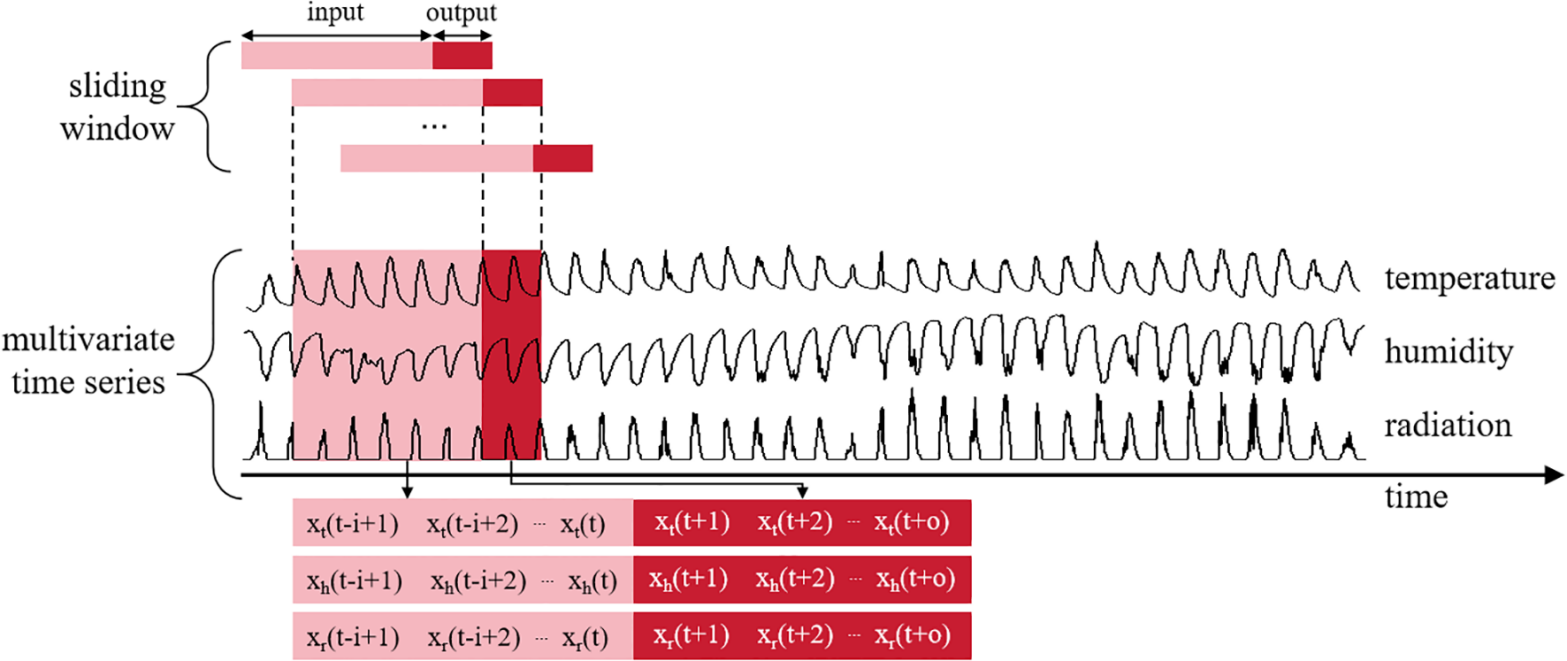
Sliding window unit structure diagram.

The SW unit systematically segments multivariate time series data to establish temporal correlations between historical observations (e.g., continuous hourly temperature, humidity, and radiation measurements) and future target values (e.g., predicted values for subsequent hours), thereby forming structured input-output pairs (Yang et al., 2023). This methodological framework helps the model explicitly learn complex cross-variable coupling relationships and multi-scale dynamic evolution patterns at different time intervals, as shown in **Figure 7**.

where *x_t_* denotes the environment variable temperature, *x_h_* denotes the environment variable humidity, and *x_r_* denotes the environment variable radiation; *i* denotes the length of the input window; *o* denotes the length of the output window; and *t* denotes the current time point.

The DP module uses WTD units for initial noise reduction processing, followed by reconstruction of the noise-reduced time series based on SW units to generate a feature matrix. This architecture enhances the model’s predictive capabilities by extracting underlying patterns in the dynamic facility environment, significantly improving the accuracy and reliability of predictions for future environmental parameters (temperature, humidity, solar radiation).

## 4 Results and analysis

### 4.1 Parameterization

The facility environment prediction model of this study was run on a hardware environment of Intel(R) Core(TM) i5-9300HF central processor and NVIDIA GeForce GTX 1650 graphics card, coded in Python 3.7, with an integrated development environment of PyCharm2024.1.2, under the framework of Pytorch1.8.1 Development and evaluation were carried out. After extensive testing, the optimal number of layers and neurons for the model was determined, with the following specific parameter settings: 2 LSTM layers, 100 hidden layer units, a learning rate of 0.001, the Adam optimizer selected, a batch size of 64, and 100 epochs.

### 4.2 Explainability and computational overhead analysis of attention mechanisms

**Figure 8:**
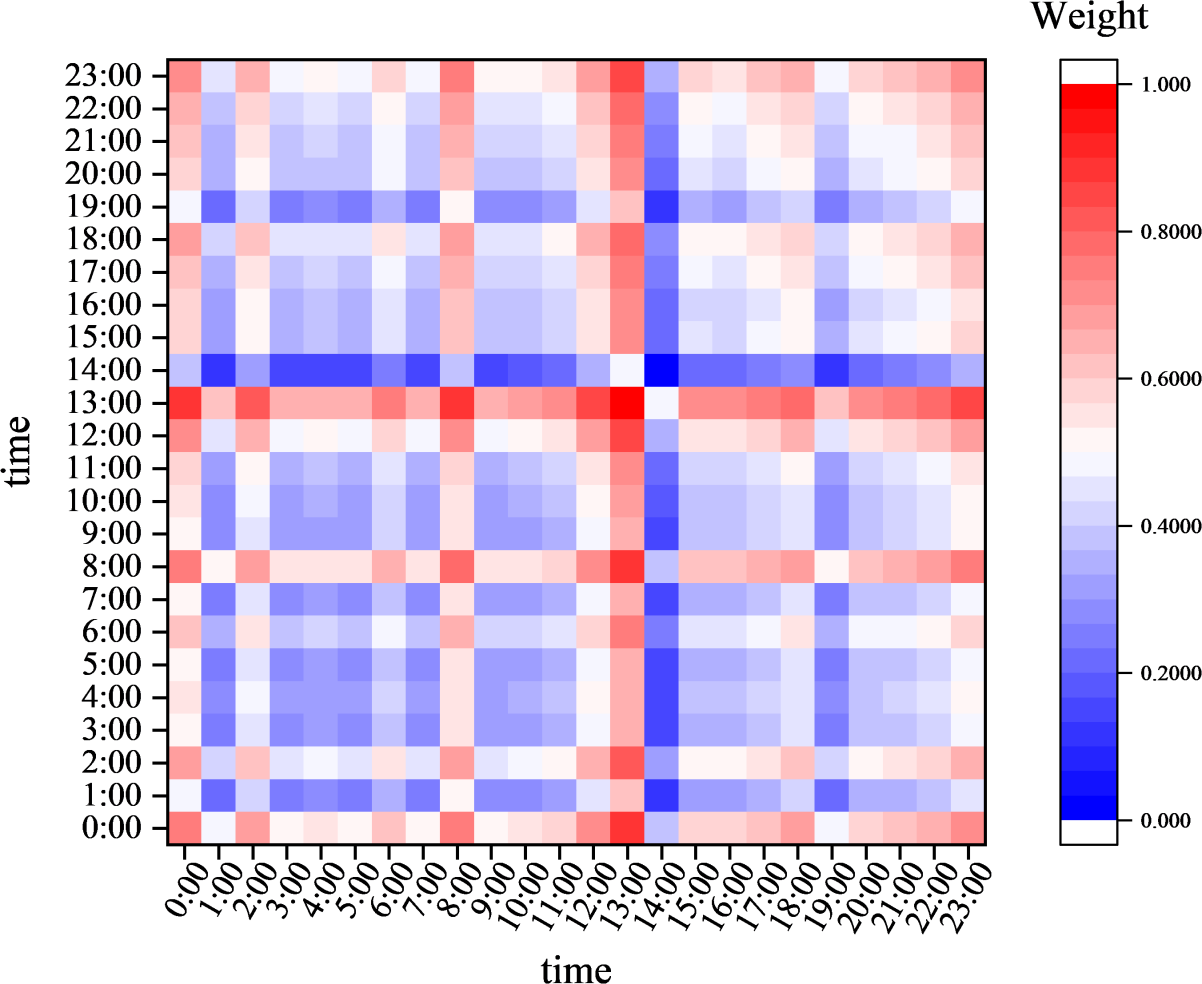
Attention weight heat map.

**Figure 8** illustrates how visualizing the distribution of attention weights across the time dimension can intuitively present the model’s dynamic attention patterns toward the input sequence. The Y-axis of the heatmap represents a single attention head, the X-axis corresponds to consecutive time steps, and the color gradient (from light yellow to dark red) reflects the model’s attention intensity toward features at each time step. The experimental results show that the prominent red regions align spatially and temporally with key feature changes, such as sudden temperature changes. This indicates that the model can effectively capture sudden temporal events. The light yellow regions correspond to periods of feature stability. Their low attention weights are statistically significantly associated with states of low information content. This visualization validates AT’s capability to dynamically focus on key temporal segments, as there is a quantifiable positive correlation between attention weights and feature importance.

The introduction of the AT module significantly improves the performance of temporal modeling, primarily due to its ability to dynamically model non-local dependencies across time steps in long sequences. While the standard LSTM model has basic temporal feature extraction capabilities, the AT module provides more refined temporal feature selection at the lightweight cost of a 4.15% increase in parameters (122,901 to 128,002). This enhancement enables the model to adaptively focus on discriminative features at critical time steps, showcasing its superior temporal pattern modeling capabilities in complex environmental prediction tasks. As shown in **Table 3** using a 24-hour temperature prediction task as an example, experimental results demonstrate that AT significantly improves the model’s accuracy in capturing nonlinear temporal dynamics by establishing global temporal context associations, thereby enhancing the robustness and reliability of prediction results. These characteristics make AT a key component for addressing long-sequence dependency issues, particularly in scenarios requiring precise modeling of temporal dynamics, such as weather forecasting and industrial equipment monitoring.

**Table 3:** Comparison and analysis of computational overhead of attention mechanisms.

Indicator	LSTM	LSTM-AT	Difference(%)
Inference time (ms)	1.4635	1.5573	6.40
Params	122901.00	128002.00	4.15
GPU memory	240.17	256.65	6.86
R^2^	0.9213	0.9566	3.83
MAE	2.1202	1.5390	37.76
RMSE	2.3673	1.7589	34.59

### 4.3 Choosing and comparing small wave functions

In wavelet threshold denoising methods, the selection of wavelet basis functions, decomposition levels, and threshold strategies are key performance factors. This study selected three representative wavelet basis functions—sym4, db4, and coif1—and three threshold strategies—universal threshold, maximum-minimum threshold, and SURE (Stein unbiased risk estimate) threshold—to systematically evaluate the impact of different parameter combinations on denoising performance. Comparative experiments were conducted at three decomposition levels (2, 4, and 6). Three objective evaluation metrics—improved signal-to-noise ratio (ASNR), root mean square error (RMSE), and correlation coefficient (CC)—were introduced to quantitatively assess denoising performance (see **Table 4**). Using temperature data as an example, experimental data show that the system achieves optimal denoising performance when using the sym4 wavelet basis function, decomposition level 2, and the minimum-maximum threshold strategy: The ASNR improves to 40.23 dB, the RMSE decreases to 0.0681, and the CC reaches 0.9999. This parameter combination significantly outperforms other experimental groups across all evaluation metrics and is therefore established as the standard configuration for subsequent research. This optimized scheme effectively preserves the feature information of the original signal while improving the signal-to-noise ratio and providing a reliable foundation for subsequent signal processing.

**Table 4:** Performance comparison of wavelet denoising under different parameter combinations.

Wavelet basis	Decomposition level	Threshold rule	ΔSNR (dB)	RMSE	CC
sym4	**2**	universal	37.50	0.0932	0.9999
		**minimax**	**40.23**	**0.0681**	**0.9999**
		sure	29.22	0.2416	0.9994
	4	universal	35.27	0.1204	0.9999
		minimax	38.34	0.0846	0.9999
		sure	22.60	0.5182	0.9973
	6	universal	34.76	0.1277	0.9998
		minimax	37.91	0.0889	0.9999
		sure	20.63	0.6497	0.9963
db4	2	universal	37.39	0.0944	0.9999
		minimax	40.16	0.0686	0.9999
		sure	28.89	0.2511	0.9994
	4	universal	35.16	0.1220	0.9998
		minimax	38.24	0.0855	0.9999
		sure	22.45	0.5271	0.9972
	6	universal	34.64	0.1295	0.9998
		minimax	37.82	0.0899	0.9999
		sure	20.60	0.6522	0.9964
coif1	2	universal	35.25	0.1208	0.9999
		minimax	37.97	0.0883	0.9999
		sure	27.69	0.2883	0.9991
	4	universal	32.97	0.1570	0.9997
		minimax	36.04	0.1103	0.9999
		sure	21.19	0.6096	0.9962
	6	universal	32.45	0.1667	0.9997
		minimax	35.61	0.1159	0.9999
		sure	19.58	0.7330	0.9952

Bolded values represent the optimal results under each evaluation metric, indicating the most effective strategy for the given task.

### 4.4 LSTM-AT-DP model ablation test

This study evaluates the effectiveness of model optimization by analyzing three key environmental parameters (temperature, humidity, and radiation). Specifically, it analyzes the individual and synergistic enhancement effects of the AT and DP modules on LSTM performance and compares the differences in prediction accuracy between isolated improvement methods and integrated improvement methods.

#### 4.4.1 Optimization evaluation of AT and DP modules on temperature prediction performance of LSTM models

As shown in the experimental data in **Table 5**, The Bi-LSTM model exhibits clear limitations in short-term forecasting. Compared to the original LSTM model, the R² value decreased by 0.31% for 6-hour forecasts and by 0.31% for 12-hour forecasts. Additionally, the MAE value increased by 0.0420 for 6-hour forecasts and by 0.0386 for 12-hour forecasts, while the RMSE value increased by 0.0612 for 6-hour forecasts and by 0.0600 for 12-hour forecasts. While the bidirectional structure improves long-term forecasting performance, it actually leads to a decline in accuracy when handling short-term forecasting tasks. For 24-hour forecasts, R² increases by 3.13%, MAE decreases by 0.5417, and RMSE decreases by 0.5309. Integrating the AT module into the LSTM model resulted in significant improvements across multiple metrics. Specifically, the R² values for 6-hour, 12-hour, and 24-hour predictions increased by 0.41%, 0.81%, and 3.53%, respectively, while the MAE decreased by 0.1333, 0.1565, and 0.5812, respectively, and the RMSE decreased by 0.0841, 0.1674, and 0.6084, respectively. These results indicate that AT significantly enhances the stability of long-term predictions. When the DP module is added, the R² values for the corresponding time intervals increased by 0.22%, 0.08%, and 3.33%, respectively, MAE decreased by 0.0445, 0.0195, and 0.5705, respectively, and RMSE decreased by 0.0448, 0.0161, and 0.5688, respectively. Notably, the DP module demonstrated particularly significant noise reduction effects in long-term forecasts. The model combining LSTM-AT-DP achieved the most significant improvements: R² increased by 1.64%, 1.33%, and 3.89%, respectively, while the MAE decreased by 0.3435, 0.1631, and 0.6272, respectively, and the root RMSE decreased by 0.3673, 0.2847, and 0.6830, respectively. It is worth noting that the R² value for the 24-hour forecast increased by 3.89%, which significantly surpasses the improvement achieved by using the AT module (3.53%) or the DP module (3.33%) alone, thereby confirming the synergistic optimization effect between the AT module and the DP module.

**Table 5:** The impact of improvements to the LSTM-AT-DP model on temperature prediction performance.

Forecast duration	Model	Bi	AT	DP	R^2^	MAE	RMSE
6h	LSTM	×	×	×	0.9559	1.5325	1.7713
	Bi-LSTM	✓	×	×	0.9528	1.5745	1.8325
	LSTM+AT	×	✓	×	0.9600	1.3992	1.6872
	LSTM+DP	×	×	✓	0.9581	1.4880	1.7265
	LSTM+AT+DP	×	✓	✓	**0.9723**	**1.1890**	**1.4040**
12h	LSTM	×	×	×	0.9540	1.5573	1.8103
	Bi-LSTM	✓	×	×	0.9509	1.5959	1.8703
	LSTM+AT	×	✓	×	0.9621	1.4008	1.6429
	LSTM+DP	×	×	✓	0.9548	1.5378	1.7942
	LSTM+AT+DP	×	✓	✓	**0.9673**	**1.3942**	**1.5256**
24h	LSTM	×	×	×	0.9213	2.1202	2.3673
	Bi-LSTM	✓	×	×	0.9526	1.5785	1.8364
	LSTM+AT	×	✓	×	0.9566	1.5390	1.7589
	LSTM+DP	×	×	✓	0.9546	1.5497	1.7985
	LSTM+AT+DP	×	✓	✓	**0.9602**	**1.4930**	**1.6843**

Bolded values represent the optimal results under each evaluation metric, indicating the most effective strategy for the given task.

#### 4.4.2 Optimization evaluation of AT and DP modules on humidity prediction performance of LSTM models

As shown in **Table 6**, the experimental results reveal distinct performance characteristics across different humidity prediction models. The Bi-LSTM model clearly demonstrates temporal dependency in its predictive capabilities. It underperforms the base LSTM model for short-term 6-hour and 12-hour forecasts, with R² reductions of 0.95% and 0.69%, respectively. This is accompanied by increased mean absolute error (MAE) of 0.5339 and 0.3363 and root mean squared error (RMSE) of 0.3742 and 0.2762, respectively. However, the Bi-LSTM model’s performance substantially improves for 24-hour predictions, achieving a 3.76% R² improvement while reducing MAE by 1.3127 and RMSE by 1.1932.In comparison, the AT-enhanced LSTM model shows consistent performance gains across all time horizons. It achieves R² improvements ranging from 0.30% to 3.88%, with MAE reductions between 0.0364 and 0.7231 and RMSE decreases between 0.1241 and 1.2346. The DP module notably improves long-term accuracy, providing a 3.05% R² increase for 24-hour forecasts while maintaining stable short-term performance. The combined LSTM-AT-DP model is the most effective solution, delivering superior performance across all prediction windows. It achieves R² improvements of 1.46%, 0.62%, and 5.53% for 6-, 12-, and 24-hour forecasts, respectively. The model’s most significant enhancement is evident in 24-hour predictions, where it reduces MAE by 1.4276 and RMSE by 1.8759. This demonstrates the synergistic benefits of integrating attention mechanisms and denoising processes. This evaluation clearly shows that, although Bi-LSTM offers long-term advantages, the LSTM-AT-DP combination provides more reliable and consistent improvements across all prediction timeframes.

**Table 6:** The impact of improvements to the LSTM-AT-DP model on humidity prediction performance.

Forecast duration	Model	Bi	AT	DP	R^2^	MAE	RMSE
6h	LSTM	×	×	×	0.9504	2.8078	4.0569
	Bi-LSTM	✓	×	×	0.9409	3.3417	4.4311
	LSTM+AT	×	✓	×	0.9571	**2.5643**	3.7751
	LSTM+DP	×	×	✓	0.9564	2.7594	3.8039
	LSTM+AT+DP	×	✓	✓	**0.9650**	2.6023	**3.4069**
12h	LSTM	×	×	×	0.9508	2.8037	4.0417
	Bi-LSTM	✓	×	×	0.9439	3.1400	4.3179
	LSTM+AT	×	✓	×	0.9538	2.7673	3.9176
	LSTM+DP	×	×	✓	0.9509	2.7711	4.0364
	LSTM+AT+DP	×	**√**	✓	**0.9570**	**2.7078**	**3.7780**
24h	LSTM	×	×	×	0.8976	4.5554	5.8316
	Bi-LSTM	✓	×	×	0.9352	3.2427	4.6384
	LSTM+AT	×	✓	×	0.9364	3.8323	4.5970
	LSTM+DP	×	×	✓	0.9281	3.6984	4.8858
	LSTM+AT+DP	×	✓	✓	**0.9529**	**3.1278**	**3.9557**

Bolded values represent the optimal results under each evaluation metric, indicating the most effective strategy for the given task.

#### 4.4.3 Optimization evaluation of AT and DP modules on radiation prediction performance of LSTM models

As shown in **Table 7**, the experimental results demonstrate significant variations in radiation prediction performance across different model architectures and time horizons. The Bi-LSTM model exhibits inconsistent performance characteristics. For six-hour predictions, it achieves performance gains, with R² increasing by 1.25 percentage points, and reducing both MAE and RMSE. However, its predictive capability deteriorates for 12-hour forecasts, showing reduced accuracy across all metrics. The model regains its predictive advantage for 24-hour forecasts, delivering an improved R² value and substantial reductions in error metrics. In comparison, the AT-enhanced LSTM model demonstrates more stable improvements across all prediction windows. It consistently increases R² values and achieves significant decreases in MAE and RMSE across 6-, 12-, and 24-hour forecasts. The DP module exhibits comparable yet slightly less pronounced enhancement effects, especially in longer-term predictions. The integrated LSTM-AT-DP model is the most robust solution, providing superior performance that surpasses the improvements of its individual components. This combined architecture achieves the highest R² values and the most substantial reductions in error metrics across all time horizons. Its 6-hour prediction performance is particularly noteworthy, as the RMSE reduction significantly outperforms what either module achieves independently. These results clearly demonstrate the complementary nature and synergistic effects of combining attention mechanisms with denoising processes for radiation prediction tasks.

**Table 7:** The impact of improvements to the LSTM-AT-DP model on radiation prediction performance.

Forecast duration	Model	Bi	AT	DP	R^2^	MAE	RMSE
6h	LSTM	×	×	×	0.9564	18.7291	32.1908
	Bi-LSTM	✓	×	×	0.9689	17.4412	27.2083
	LSTM+AT	×	✓	×	0.9845	11.7840	19.2188
	LSTM+DP	×	×	✓	0.9784	12.2100	22.6659
	LSTM+AT+DP	×	✓	✓	**0.9868**	**11.2566**	**17.6814**
12h	LSTM	×	×	×	0.9569	18.6673	31.9918
	Bi-LSTM	✓	×	×	0.9560	18.8199	32.3436
	LSTM+AT	×	✓	×	0.9812	13.1067	21.1537
	LSTM+DP	×	×	✓	0.9780	14.5867	22.8860
	LSTM+AT+DP	×	✓	✓	**0.9850**	**12.1266**	**18.8853**
24h	LSTM	×	×	×	0.9555	18.8610	32.5265
	Bi-LSTM	✓	×	×	0.9664	17.7508	28.2504
	LSTM+AT	×	✓	×	0.9802	13.5160	21.7183
	LSTM+DP	×	×	✓	0.9725	16.1093	25.5568
	LSTM+AT+DP	×	✓	✓	**0.9839**	**12.5898**	**19.5745**

Bolded values represent the optimal results under each evaluation metric, indicating the most effective strategy for the given task.

### 4.5 Exploration of the performance of the LSTM-AT-DP model in comparison with the classical model

To validate the efficacy of the enhanced LSTM-AT-DP model, this study performed comparative experiments against several classical baseline models, including RNN, LSTM, and GRU architectures. The experimental results demonstrate that our proposed model achieves superior performance across all evaluation metrics.

#### 4.5.1 Comparison and evaluation of temperature prediction performance of LSTM-AT-DP model and classical models

**Figure 9:**
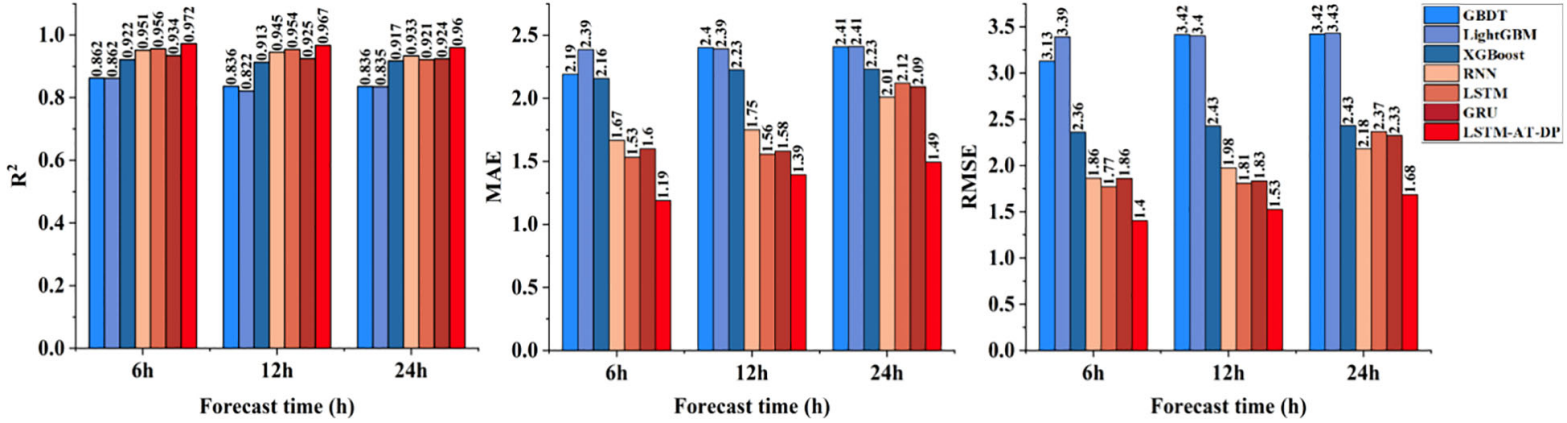
Comparison of the temperature prediction performance of the LSTM-AT-DP model and the classical model.

As shown in the data in **Figure 9**, the LSTM-AT-DP model achieved significant R² values of 0.9723, 0.9673, and 0.9602 for 6-hour, 12-hour, and 24-hour predictions, respectively, representing improvements of 1.7% to 4.2% compared to the traditional LSTM model. Additionally, the model achieved significant reductions in MAE and RMSE, decreasing by 22.4% to 29.6% and 20.7% to 28.8%, respectively. Notably, the 24-hour prediction performance of the LSTM-AT-DP model achieved an MAE of 1.4930, significantly outperforming the LSTM (2.1202) and GRU (2.0924) benchmarks. This performance improvement can be attributed to the model’s innovative architecture: the AT module mitigates gradient decay issues in long-term predictions by dynamically focusing on key temporal features, while the DP module provides robust multi-scale signal processing capabilities. The synergistic integration of these components enables the LSTM-AT-DP model to achieve higher accuracy and stability in temperature forecasting tasks.

#### 4.5.2 Comparison and evaluation of humidity prediction performance of LSTM-AT-DP model and classical models

**Figure 10:**
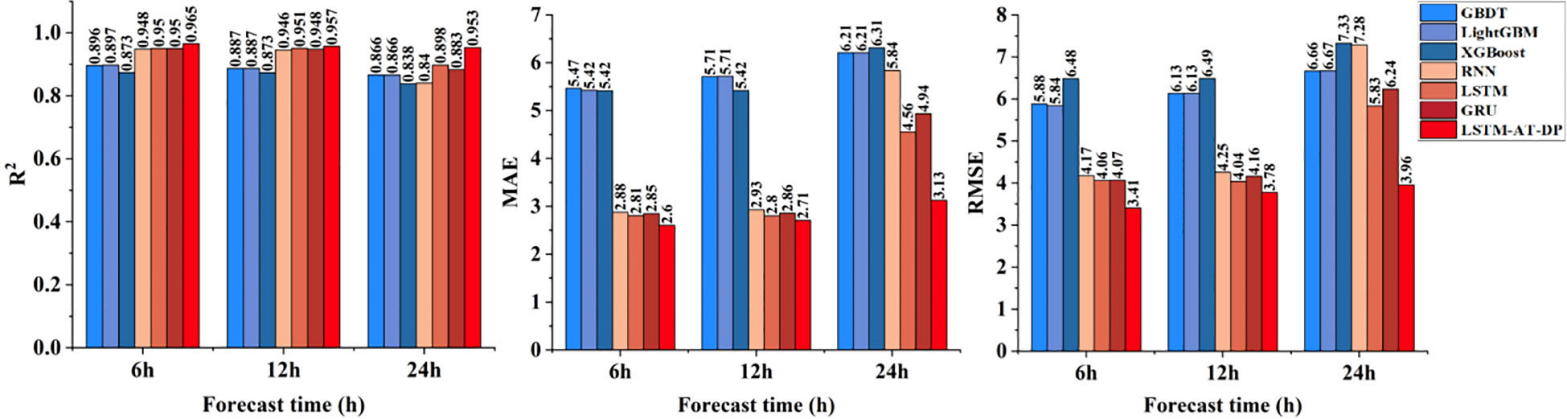
Comparison of the humidity prediction performance of the LSTM-AT-DP model and the classical model.

**Figure 10** shows a comprehensive performance comparison of various models in a humidity prediction task characterized by highly nonlinear dynamics and significant noise interference. The LSTM-AT-DP model performs exceptionally well, achieving an R² value of 0.9529 for 24-hour predictions, representing a 6.2% improvement over the baseline LSTM model’s R² value of 0.8976. Notably, the MAE is reduced to 3.1278, a significant 31.4% decrease compared to the LSTM baseline model. Comparing the prediction curves shows that the LSTM-AT-DP model exhibits significantly reduced prediction fluctuations in low signal-to-noise ratio regions, confirming the DP module’s exceptional noise separation capability. These results collectively indicate that the LSTM-AT-DP model possesses a clear advantage in handling noisy humidity prediction scenarios, achieving more accurate predictions of humidity changes through its effective noise suppression and feature extraction mechanisms.

#### 4.5.3 Comparison and evaluation of radiation prediction performance of LSTM-AT-DP model and classical models

**Figure 11:**
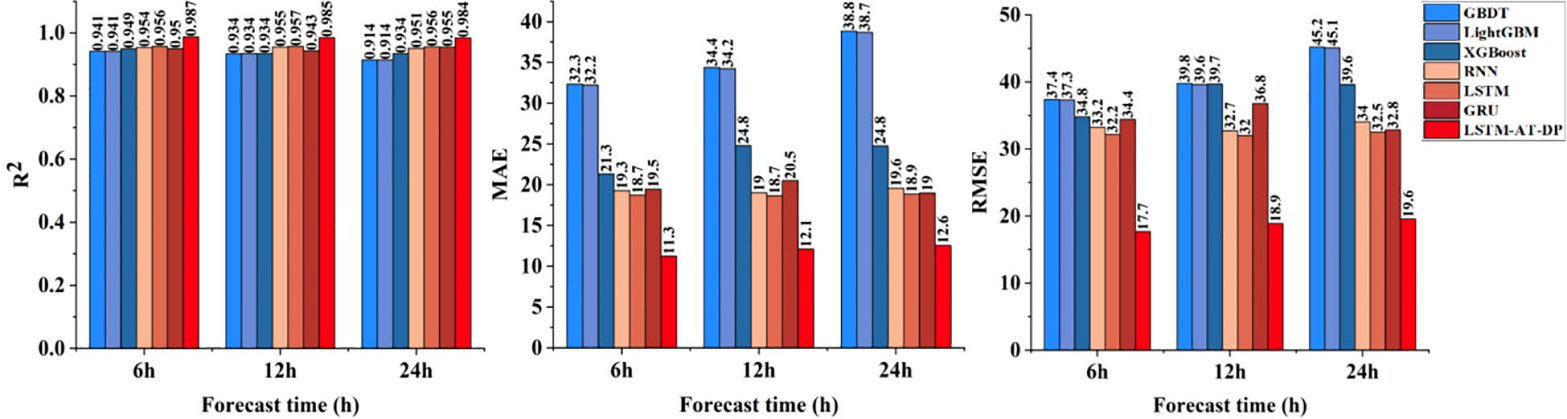
Comparison of the radiation prediction performance of the LSTM-AT-DP model and the classical model.

**Figure 11** demonstrates the outstanding performance of the LSTM-AT-DP model in radiation prediction tasks with frequent mutation characteristics. The model achieved an excellent R² value of 0.9868 in 6-hour predictions, with a corresponding MAE of 11.2566, representing a significant reduction of 39.9% compared to the MAE (18.7291) of the LSTM baseline model. Detailed curve analysis shows that the LSTM-AT-DP model consistently achieves prediction errors below 5% during the midday radiation peak period, significantly outperforming traditional models (error range of 8% to 12%). This performance advantage stems from two core mechanisms: the AT module enhances feature weights during sudden changes, and the DP module improves transient response accuracy through advanced high-frequency signal decomposition techniques. For 24-hour predictions, the model’s MAE is 12.5898, which is 49.2% lower than XGBoost’s MAE (24.7675), and the R² is improved by 5.4%, further validating the superiority of this deep learning architecture in spatio-temporal nonlinear modeling.

## 5 Discussion

**Figure 12:**
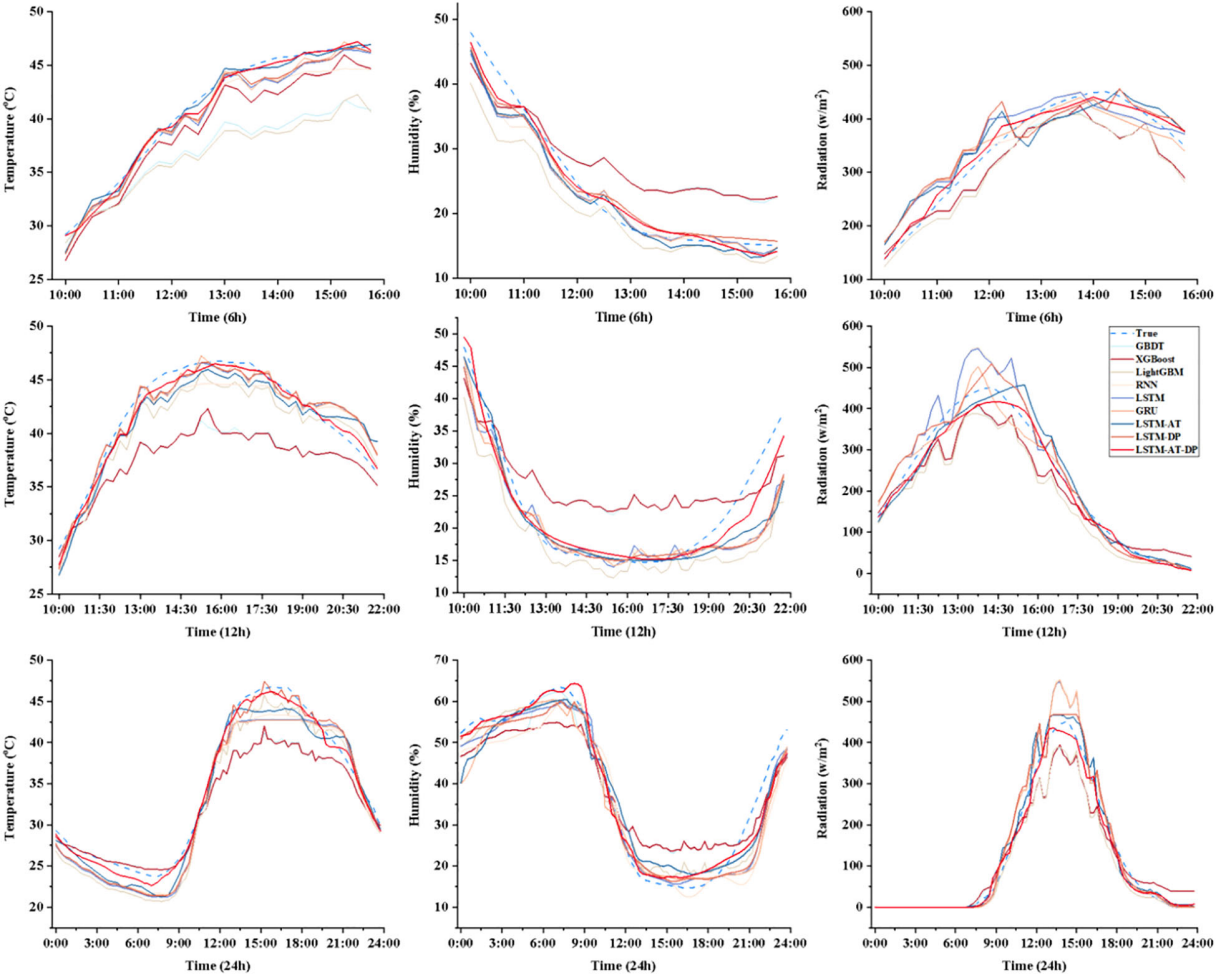
Comparison of prediction results in multi-model environments.

**Figure 12** shows a comprehensive comparison of the predictive performance of the proposed LSTM-AT-DP model and traditional models in facility environments under different environmental parameters (temperature, humidity, radiation) and different forecast lead times (6 hours, 12 hours, 24 hours). Through a systematic analysis of **Figures 8**-**11**, the LSTM-AT-DP model demonstrates significant advantages, specifically as follows:

The LSTM-AT-DP model demonstrates excellent temporal stability when the prediction time is extended from 6-hour to 24-hour, with only a minimal decline in performance. In terms of temperature prediction, the model exhibits excellent consistency, with an R² value fluctuation of only 0.0121, which is 63.5% and 55.8% higher than the LSTM model (0.0346) and GRU model (0.0274), respectively. In humidity prediction, the model achieves a 24-hour MAE of 3.1278, an improvement of 46.4% over RNN (5.8399), while maintaining an RMSE of 3.9557, which is 32.2% lower than LSTM’s 5.8316. In radiation forecasting, the model demonstrated near-perfect temporal consistency, with R² values for 6 hour (0.9868) and 24 hour (0.9839) predictions differing by less than 0.3%, significantly outperforming the 1.6% performance degradation of the XGBoost model. These results collectively validate the model’s unprecedented stability in multi-variable long-term environmental forecasting.

The LSTM-AT-DP model demonstrates superior cross-variable prediction performance across all three environmental parameters (temperature, humidity, and radiation). For 24-hour predictions, the model achieves MAE values of 1.4930 (temperature) and 3.1278 (humidity), with a remarkably small inter-variable difference of merely 1.6348 significantly lower than the 2.4352 observed in the baseline LSTM model. This substantial 32.9% reduction in performance variance clearly indicates the model’s enhanced capability to balance the learning of coupling relationships between different environmental factors. In radiation forecasting, the model’s 12-hour MAE of 12.1266 represents a 51.1% improvement over XGBoost (24.7902), while its RMSE of 18.8853 is only 47.6% of conventional models. These results provide compelling evidence that the LSTM-AT-DP architecture effectively captures high-frequency transient components while maintaining both high efficiency and accuracy in multi-variable prediction tasks, owing to its sophisticated feature extraction and noise suppression mechanisms.

The LSTM-AT-DP model demonstrates significant advantages in modeling different environmental factors. In 24-hour radiation forecasts, the model achieved a MAE of 12.5898, a 49.2% reduction compared to XGBoost (24.7675), while increasing the coefficient of determination R² by 5.4 percentage points. This significant improvement clearly demonstrates the superior ability of deep learning architectures to capture the inherent spatio-temporal nonlinear relationships in complex radiation data. In humidity prediction, the model achieved an R² value of 0.9529 within 24 hours, which is 7.9% higher than GRU (0.8829), while reducing the MAE value by 36.7%. These improvements clearly indicate that the integration of the AT module and DP module effectively enhances feature selection and noise suppression capabilities, enabling more precise identification of key humidity patterns while minimizing interference from noise signals, thereby significantly improving prediction accuracy.

Data analysis indicates that the DP module significantly improves data quality through the synergistic integration of the WTD unit and SW unit. The WTD unit utilizes the time-frequency localization characteristics of the sym4 wavelet basis function to achieve precise separation of signals and noise in facility environmental monitoring data, particularly excelling in suppressing high-frequency noise components. Experimental results show that in radiation prediction tasks, the independent DP module achieved error reductions of 6.5191 W/m² (MAE) and 9.5249 W/m² (RMSE), confirming its dual capabilities in noise elimination and high-frequency signal retention. In 24-hour temperature forecasts, the DP module increased the R² coefficient by 3.33% while reducing MAE and RMSE by 0.5705 and 0.5688, respectively. These metrics collectively validate the module’s exceptional performance in handling non-stationary signals and its robust capability to optimize data quality. The SW unit operates by dividing continuous time series into a structured mapping from historical states to future targets. This architectural approach enables the LSTM network to capture the systematic spatiotemporal dynamics of environmental processes rather than discrete-time features, significantly enhancing the model’s ability to learn complex spatiotemporal dependencies among environmental variables.

By integrating the AT module, the model can dynamically adjust feature weights at critical time points, significantly enhancing its ability to detect sudden environmental events. In experiments verifying humidity forecasts, the AT module reduced the MAE of 24-hour predictions by 36.7% and increased the R² by 5.53%. These metrics confirm the module’s dual functionality: it can both precisely allocate attention to key features and effectively suppress noise, thereby improving prediction accuracy. The AT module automatically enhances feature extraction during crop-sensitive phenological periods by calculating the similarity between Q and K. This architecture ensures that prediction performance remains robust even under low signal-to-noise ratio conditions. Notably, in the 24-hour humidity forecast task, the model incorporating the AT module achieved an MAE of just 3.1278% RH, representing a 36.7% improvement over the traditional GRU model. This comparative advantage fully demonstrates the effectiveness of the AT module in strategic information focusing and noise suppression.

The synergistic integration of the DP module and the AT module has effectively broken through the bottleneck of declining accuracy in long-term forecasts. The DP module ensures high signal-to-noise ratio input data, while the AT module dynamically enhances feature representation capabilities at critical time points through adaptive weight allocation. This dual optimization strategy significantly alleviates the error propagation issue in sequence prediction. Experimental results show that compared to the baseline LSTM model, the integrated model achieves R² improvements of 3.89%, 5.53%, and 2.84% in 24-hour temperature, humidity, and radiation forecasts, respectively, while reducing MAE by 29.6% to 39.9%. Notably, the model’s ability to detect sudden events has significantly improved: for 6-hour radiation forecast peaks, the MAE percentage has dropped to 3.2%–4.8%, representing a significant improvement over the 8%–12% range observed in traditional models. These findings not only validate the accuracy improvements achieved through the synergistic effects of DP-AT but also highlight the model’s stability and ability to capture sudden events.

The multi-factor time series modeling method establishes a unified representation of the coupled relationships between temperature, humidity, and radiation through multivariate reconstruction and deep feature extraction driven by the DP module based on LSTM-AT, thereby demonstrating outstanding performance. The model maintains excellent stability, with R² values for all three factors remaining above 0.95 in both 6-hour and 24-hour forecasts. Compared to the baseline model, cross-factor performance variability is significantly reduced (LSTM-AT-DP exhibits 1.46% variability in humidity forecasts, while GRU shows 7.9%). Under low signal-to-noise ratio conditions, the synergistic effect of the DP module and AT module ensures robust performance: the DP module maintains data reliability by effectively suppressing noise, while the AT module dynamically optimizes feature weights. This combination achieves an MAE value as low as 3.1278% RH in 24-hour humidity forecasts, representing a 36.7% improvement over GRU. These results validate the model’s ability to balance learning cross-factor coupling relationships and capturing high-frequency mutations. In radiation prediction, the LSTM-AT-DP model achieved an R² of 0.9868 and a 6-hour MAE of 11.2566 W/m², representing a 39.9% improvement over the LSTM model. This indicates that the AT module focuses more on transient events, while the DP module enhances the resolution of high-frequency signal components through advanced decomposition techniques.

## 6 Conclusion

This study proposes a model based on LSTM-AT-DP, which achieves significant improvements in accuracy and stability in time series prediction of multiple factors (temperature, humidity, and radiation) in controlled agricultural environments through the collaborative optimization of the DP module and the AT module. The DP module effectively removes data noise and improves data quality using WTD and SW units, thereby providing structurally optimized inputs for subsequent modeling. Experimental results show that in the temperature prediction task, the independent DP module improves the 24-hour prediction R² metric by 3.33%, confirming its exceptional adaptability to non-stationary signals. Meanwhile, the AT module amplifies feature representations at critical time points through dynamic weight allocation, significantly enhancing the model’s ability to detect sudden environmental changes. This effect was quantitatively verified in a humidity prediction task, where the integration of the AT module reduced the 24-hour MAE metric by 36.7%, fully demonstrating its accuracy in focusing on time-sensitive information.

Comprehensive experimental evaluations demonstrate that the proposed LSTM-AT-DP architecture outperforms traditional models across all evaluation metrics (R², MAE, and RMSE), particularly in long-term forecasting tasks. Specifically, the model achieved R² values of 0.9602 for temperature, 0.9529 for humidity, and 0.9839 for radiation in 24-hour forecasts, representing improvements of 3.89%, 5.53%, and 2.84%, respectively, compared to the baseline LSTM model. More notably, the model achieved significant error reduction, with the MAE for temperature prediction decreasing from 2.1202 to 1.4930 (a reduction of 29.6%) and the RMSE decreasing from 2.3673 to 1.6843 (a reduction of 28.9%). These quantitative results clearly demonstrate the model’s exceptional ability to handle multi-factor coupling relationships and noise interference in controlled agricultural environments. Especially under harsh conditions with low signal-to-noise ratios, the model still maintains robust prediction accuracy—this finding provides empirical validation for the synergistic optimization between the noise suppression DP module and the feature enhancement AT module.

In summary, the LSTM-AT-DP model provides a solid technical foundation for precise environmental control in facility agriculture. The model’s superior performance in terms of prediction accuracy (parameter-average R² improved by 4.09%), temporal stability (MAE reduced by 39.9%), and transient event detection capability (peak error reduced to 3.2–4.8%) has been rigorously validated through comprehensive experimental data. In the future, the application of this model can be further expanded to different types of facilities and crop varieties to verify its universality and promotional value. At the same time, more advanced deep learning technologies and data processing methods can be explored to further improve the model’s predictive performance and practicality.

## Data Availability

The raw data supporting the conclusions of this article will be made available by the authors, without undue reservation.
